# Clinical Relevance of Troponin T Profile Following Cardiac Surgery

**DOI:** 10.3389/fcvm.2018.00182

**Published:** 2018-12-13

**Authors:** Hendrik T. Tevaearai Stahel, Peter D. Do, Jeremias Bendicht Klaus, Brigitta Gahl, Didier Locca, Volkhard Göber, Thierry P. Carrel

**Affiliations:** ^1^Department of Cardiovascular Surgery, Bern University Hospital and University of Bern, Bern, Switzerland; ^2^Institute of Radiology, Inselspital, Bern University Hospital and University of Bern, Bern, Switzerland; ^3^Department of Cardiology, Barts Heart Center, Barts Health NHS Trust, London, United Kingdom; ^4^William Harvey Institute, Queen Mary University London, United Kingdom

**Keywords:** cardiac surgery, CABG, troponin, cardiac biomarkers, complications

## Abstract

**Background:** Peak post-operative cardiac troponin T (cTnT) independently predicts mid- and long-term outcome of cardiac surgery patients. A few studies however have reported two peaks of cTnT over the first 48–72 h following myocardial reperfusion. The aim of the current study was to better understand underlying reasons of these different cTnT profiles and their possible relevance in terms of clinical outcome.

**Methods:** All consecutive adult cardiac surgical procedures performed with an extra-corporeal circulation during a >6 years period were retrospectively evaluated. Patients with a myocardial infarction (MI) < 8 days were excluded. cTnT profile of patients with at least one value ≥1 ng/mL value were categorized according to the time occurrence of the peak value. Univariable and multivariable analysis were performed to identify factors influencing early vs. late increase of cTnT values, and to verify the correlation of early vs. late increase with clinical outcome.

**Results:** Data of 5,146 patients were retrieved from our prospectively managed registry. From 953 with at least one cTnT value ≥1 ng/mL, peak occurred ≤ 6 h (*n* = 22), >6 to ≤ 12 h (*n* = 366), >12 to ≤ 18 h (*n* = 176), >18 to ≤ 24 h (171), >24 h (218). Age (OR: 1.023; CI: 1.016–1.030) and isolated CABG (OR: 1.779; CI: 1.114–2.839) were independent predictors of a late increase of cTnT over a limit of 1 ng/ml (*p* < 0.05), whereas isolated valve procedures (OR: 0.685; CI: 0.471–0.998) and cross-clamp duration (OR: 0.993; CI: 0.990–0.997) independently predicted an early elevation (*p* < 0.05). Delayed elevation as opposed to early elevation correlated with a higher rate of post-operative complications including MI (19.8 vs. 7.2%), new renal insufficiency (16.3 vs. 6.7%), MACCE (32.0 vs. 15.5%), or death (7.4 vs. 4.4%).

**Conclusion:** Profile of cTnT elevation following cardiac surgery depends on patients' intrinsic factors, type of surgery and duration of cross-clamp time. Delayed increase is of higher clinically relevance than prompt post-operative elevation.

## Introduction

Biomarkers of myocardial injury invariably increase following cardiac surgical interventions. Reasons essentially include the direct surgical trauma to the cardiac tissue, the quality of the myocardial protection, the type of surgery (1, 2), the duration of ischemic cross clamp time (3–7), as well as conditions related to the patient himself, such as the renal function (8–11). Several studies have attempted to define biomarkers' cut-off values to predict outcome after cardiac surgery (1, 12–15). The results appear however inconsistent, partially because of inter-institutional variations in terms of overall patients' management, but also because of variations in methodological approaches. In addition and maybe more importantly, these biomarkers are not assessed in a continuous manner but only at certain time points. In other words, our current practice does not allow to precisely measure the peak of release. As a consequence, the use of cut-off values can currently not be implemented in routine practice (16). Recent guidelines on the diagnosis of perioperative myocardial infarction (PMI) have even warned about misinterpretation (17, 18).

Cardiac troponin T (cTnT), because of its high sensitivity, has lately been increasingly investigated as possible clinically relevant marker of perioperative myocardial damage. But like for other biomarkers, the difficulty in assessing its clinical relevance comes from the fact that the timing of peak elevation is not known. It has also been previously suggested that the post-operative cTnT evolution is in fact bimodal with an early peak occurring at around 6 h and a later peak occurring at 24–36 h (14, 19–21). Therefore, it would be advantageous to better understand the factors that influence the profile of post-operative cTnT evolution and possibly identify groups of patients based on their expected peak of TnT release. In addition, it is of interest to clarify if a certain cTnT elevation has the same clinical significance if it occurs rapidly after the end of surgery of later during the post-operative evolution.

In the current analysis of a prospectively collected population of several thousands of cardiac surgery patients, we first intended to characterize the post-operative profiles of cTnT evolution and verify their possible dependence on patients- or surgery-related factors. Additionally, we aimed at evaluating the clinical relevance of these profiles in terms of complications, such as PMI, stroke and death.

## Methods

### Study Design and Patients Population

**Figure 1:**
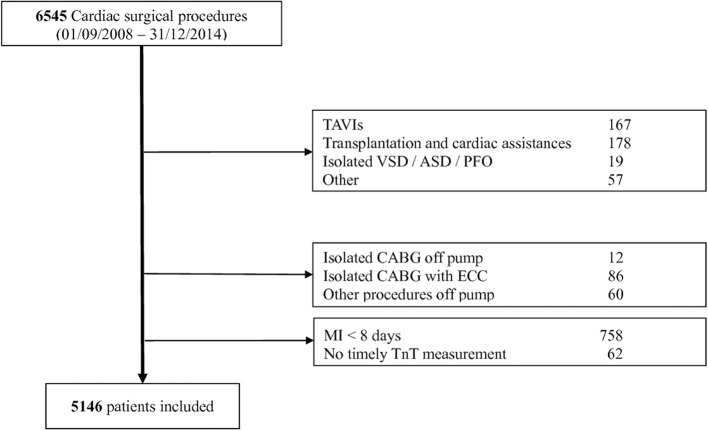
Flowchart of patients' selection.

Retrospective single center study including all consecutive adult patients undergoing a cardiac surgery procedure, between September 2008 and December 2014, and identified from a prospectively updated registry (Intellect 1.7, Dendrite Clinical Systems, Henley-on-Thames, UK). TAVIs, heart transplantation, procedures related to a cardiac assist system, isolated septal procedures as well as other procedures, such as trauma, embolectomy, pace-makers were excluded. For the sake of homogeneity of the study groups and because isolated CABG procedures are practically always performed with a mini extra-corporeal circuit (MECC) in our institution, the few CABG procedures performed with regular ECC or off-pump were excluded. Finally, patients with a myocardial infarction (MI) diagnosed <8 days before surgery were also excluded in order to guarantee a normal basal cTnT level (Figure 1). The cantonal ethics committee approved the current analysis as part of our regularly performed quality control. All patients provided informed consent regarding the collection of their data in our registry and their pseudonymization before analysis. All raw data supporting the conclusions of this manuscript can be made available by the authors, without undue reservation, to any qualified researcher.

### Surgical Procedures

Surgical procedures were standardized and performed typically via a full sternotomy using a mini extracorporeal circulation (MECC) for isolated CABG or a standard ECC for other procedures, and moderate systemic hypothermia (32°C). A single shot low volume (100 ml) cardioplegia (Cardioplexol™, Laboratory Dr. G. Bichsel AG, Unterseen, Switzerland) was used for isolated CABG procedures and repeated after 45–60 min in case of prolonged cross-clamp time. In other procedures, the cardiac arrest was induced with 100 ml Cardioplexol™ and followed every 20 min with blood cardioplegia. Anesthesia was also standardized and included the use of fentanyl, midazolam and isoflurane.

### Biomarkers Assessment

cTnT and CK-MB were routinely assessed 2–3 times during the first 24 post-operative hours. If clinically indicated, cTnT was further followed until the values started decreasing. Both TnT and CK-MB were measured using an electrochemoluminescent enzyme immunoassay on a Modular analytics E170 platform (Roche Diagnostics, Mannheim, Germany). The upper limit of normal was 0.014 ng/ml for the cTnT assay. For the purpose of the study, the time of cTnT assessment was calculated from the time at the end of the cardiopulmonary bypass and peak values of each patient were then categorized in one of the following categories: ≤6, >6 to ≤12, >12 to ≤18, >18 to ≤24, >24 h. Unfortunately, cTnT was not systematically recorded prior to surgery and a baseline value cannot be provided.

### Data Acquisition and Definitions

All variables were retrieved from our institutional prospectively maintained electronic registry, checked for completeness and plausibility by two independent data managers, and if needed completed or corrected with the aid of clinical records. The diagnosis of a perioperative MI was made according to the *Third Universal Definition of Myocardial Infarction* and the SYNTAX trial as previously described (22). MACCE, major adverse cardiovascular or cerebrovascular event, was defined as a composite of all-cause mortality, MI, or stroke. Stroke was defined by episodes of neurological dysfunction related to a focal cerebral infarction that was confirmed by imaging. A new renal insufficiency was defined by a new need of dialysis or, in patients with preoperative creatinine < 2 mg/dl, by a post-operative value of creatinine that increased above 2 mg/dl and reached at least twice the preoperative value.

### Statistics

Continuous variables are summarized as mean ± SD if normally distributed or as geometric mean with reference range, and comparisons were made using linear or Poisson regression, respectively. Dichotomous variables are expressed in absolute numbers and percentages, and comparisons were made using Fishers exact test. Non-parametric tests for trend were used to assess the relationships between the five different time intervals with respect to pre-, intra-, and post-operative data. Multivariable logistic regression modeling was used to assess independent associations between predictors and the period of peak cTnT occurrence. All tests were two-sided and *p* < 0.05 were considered statistically significant. All statistical analysis were performed with Stata (version 12, Stat Corp, College Station, Texas, USA).

## Results

A total of 6,545 patients were considered (Figure 1) from which 1,399 were excluded, mostly due to a recent MI (*n* = 758; 11.6%). The study group consisted then of 5,146 patients of whom 953 (18.5%) presented at least one post-operative cTnT value ≥1 ng/mL. The characteristics of these patients and their difference with those whose cTnT value remained under 1 ng/ml are presented in Table 1A (patients characteristics), Table 1B (procedure characteristics), and Table 1C (post-operative outcomes).

**Table 1A:** Characteristics of patients with elevated vs. “normal” post-operative cTnT values.

	**cTnT < 1 ng/mL (*n* = 4,193)**	**cTnT ≥1 ng/mL (*n* = 953)**	***p***
Age (y)	66.0 ± 11.6	65.0 ± 12.9	0.020
Female gender (*n*, %)	1,129 (26.9)	299 (31.4)	0.006
Body mass index (kg/m^2^)	27.5 ± 6.8	27.0 ± 7.8	0.088
Diabetes (*n*, %)	880 (21.0)	177 (18.6)	0.000
Insulin therapy (*n*, %)	288 (6.9)	72 (7.6)	0.045
Current smoker (*n*, %)	2,220 (52.9)	460 (48.3)	0.000
Hypertension (*n*, %)	3,103 (74.0)	680 (71.4)	0.002
Dyslipidemia (*n*, %)	2,887 (68.9)	534 (56.0)	0.000
Previous PCI (*n*, %)	506 (12.1)	89 (9.3)	0.032
Previous cardiac surgery (*n*, %)	238 (5.7)	129 (13.5)	0.006
Last pre-operative creatinine (μmol/L)	87.2 ± 43.8	107.7 ± 96.9	0.000
Number if diseased cor. art.			0.000
0 coronary artery disease (*n*, %)	1,753 (41.8)	526 (55.2)	
1 coronary artery disease (*n*, %)	287 (6.8)	68 (7.1)	
2 coronary artery disease (*n*, %)	454 (10.8)	80 (8.4)	
3 coronary artery disease (*n*, %)	1,699 (40.5)	279 (29.3)	
Left main coronary artery disease (*n*, %)	442 (10.5)	63 (6.6)	0.049
Ejection fraction (*n*, %)	58.2 ± 11.6	56.5 ± 11.7	0.000
CCS III or IV (*n*, %)	742 (17.7)	141 (14.8)	0.032
NYHA III or IV (*n*, %)	1,051 (25.1)	343 (36.0)	0.000
Urgency			0.000
Emergency (*n*, %)	235 (5.6)	133 (14.0)	
Urgent (*n*, %)	359 (8.6)	92 (9.7)	
Logistic EuroSCORE	8.4 ± 11.1	14.8 ± 17.5	0.000

*PCI, percutaneous coronary intervention; CCS, Canadian Cardiovascular Society classification for angina pectoris; NYHA, New York Heart Association classification for cardiac insufficiency*.

**Table 1B:** Procedure characteristics of patients with elevated vs. “normal” post-operative cTnT values.

	**cTnT < 1 ng/mL (*n* = 4,193)**	**cTnT ≥1 ng/mL (*n* = 953)**	***p***
Number of distal anastomoses	2.8 ± 1.4	2.6 ± 1.6	0.000
Duration of operation (min)	209.9 ± 60.6	259.5 ± 88.9	0.000
ECC time (min)	88.8 ± 37.8	137.2 ± 61.1	0.000
Cross clamp time (min)	59.6 ± 27.4	89.9 ± 40.3	0.000
Defibrillation (*n*, %)	968 (23.1)	346 (36.3)	0.000
Grouping of operations:			0.000
Stand. isol. CABG (*n*, %)	1,658 (39.5)	164 (17.2)	
CABG & valve (*n*, %)	586 (14.0)	192 (20.1)	
Isol. aortic valve (*n*, %)	715 (17.1)	89 (9.3)	
Isol. mitral valve (*n*, %)	250 (6.0)	96 (10.1)	
Other (*n*, %)	984 (23.5)	412 (43.2)	

*CABG, coronary artery bypass grafting; ECC, extra corporeal circulation*.

**Table 1C:** Post-operative outcomes of patients with elevated vs. “normal” post-operative cTnT values.

	**cTnT < 1 ng/mL (*n* = 4,193)**	**cTnT ≥1 ng/mL (*n* = 953)**	***p***
Max. cTnT value (ng/mL)^*^	0.4 (0.4–0.4)	1.9 (1.8–2.0)	0.000
Max. CK-MB value (μg/L)^*^	16.0 (15.8–16.3)	56.4 (53.3–59.6)	0.000
Myocardial infarction (*n*, %)	13 (0.3)	140 (14.7)	0.000
Resuscitation (*n*, %)	20 (0.5)	38 (4.0)	0.000
Stroke (*n*, %)	135 (3.2)	75 (7.9)	0.000
Death (*n*, %)	24 (0.6)	59 (6.2)	0.000
MACCE (*n*, %)	165 (3.9)	241 (25.3)	0.000
ICU stay (d)^*^	1.2 (1.2–1.2)	1.7 (1.6–1.8)	0.000
ICU stay >48 h (*n*, %)	581 (13.9)	338 (35.5)	0.000
New AF (*n*, %)	1,002 (23.9)	248 (26.0)	0.130
Max. creatinine value (μmol/L)^*^	84.2 (83.2–85.2)	100.2 (96.9–103.6)	0.000
New renal insufficiency (*n*, %)	121 (2.9)	118 (12.4)	0.000
Length of stay (d)^*^	8.8 (8.7–8.9)	11.2 (10.7–11.7)	0.000
1 year mortality (*n*, %)	79 (1.9)	82 (8.6)	0.000
Mortality during FU (*n*, %)	168 (4.0)	114 (12.0)	0.000

* *Calculations are based on the geometric mean and reference ranges. MACCE, major adverse cardiovascular or cerebrovascular event (as a composite of all-cause mortality, myocardial infarction, or stroke); ICU, intensive care unit*.

**Figure 2:**
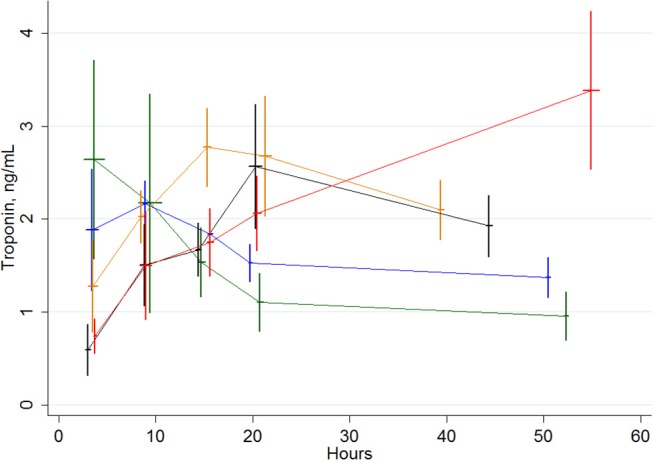
Evolution of cTnT values in subgroups of patients with a peak of cTnT of ≥1 ng/mL and occurring ≤ 6 h (green curve), between >6 to ≤ 12 h (blue curve), between >12 and ≤ 18 h (orange curve), between >18 and ≤ 24 h (black curve) and >24 h (red curve). Vertical bars are standard deviations for the cTnT values. Horizontal bars are standard deviations for the time of blood sampling.

The profile of cTnT evolution critically varied depending on the period when the peak occurred (Figure 2). When considering only patients with at least one value ≥1 ng/mL, a few patients' (Table 2A) and procedures' (Table 2B) characteristics also significantly varied with the period of cTnT peak occurrence. Age, diabetes, preoperative renal function, coronary artery disease and a history of previous cardiac intervention were all intrinsic parameters that significantly differed between patients with an early vs. late elevation of cTnT. In addition, isolated CABG, isolated valve procedures and prolonged ECC are operative parameters that were also associated with different post-operative cTnT profiles. Interestingly, gender, ejection fraction, NYHA category and urgency/emergency, although differently represented between patients with a max cTnT ≥1 ng/mL, were not associated with different post-operative cTnT profiles.

**Table 2A:** Patient characteristics according to the post-operative period of cTnT peak occurrence in patients with elevated cTnT course (≥1 ng/mL).

	**Period of cTnT peak occurrence**				
	**≤6 h**	**>6 to ≤12 h**	**>12 to ≤18 h**	**>18 to ≤24 h**	**>24 h**	***p***
	**(*n* = 22)**	**(*n* = 366)**	**(*n* = 176)**	**(*n* = 171)**	**(*n* = 218)**
Age (y)	59.1 ± 18.2	62.5 ± 14.2	63.9 ± 12.9	67.5 ± 10.3	68.9 ± 10.4	0.000
Female gender (*n*, %)	3 (13.6)	117 (32.0)	56 (31.8)	62 (36.3)	61 (28.0)	0.742
Diabetes (*n*, %)	6 (27.3)	50 (13.7)	31 (17.6)	36 (21.1)	54 (24.8)	0.007
Current smoker (*n*, %)	12 (54.5)	170 (46.4)	81 (46.0)	96 (56.1)	101 (46.3)	0.995
Hypertension (*n*, %)	9 (40.9)	243 (66.4)	124 (70.5)	141 (82.5)	163 (74.8)	0.000
Dyslipidemia (*n*, %)	10 (45.5)	183 (50.0)	95 (54.0)	107 (62.6)	139 (63.8)	0.026
Previous PCI (*n*, %)	0 (0.0)	20 (5.5)	12 (6.8)	25 (14.6)	32 (14.7)	0.000
Previous cardiac surgery (*n*, %)	3 (13.6)	52 (14.2)	29 (16.5)	19 (11.1)	26 (11.9)	0.031
Last pre-operative creatinine (μmol/L)	89.0 ± 22.1	96.6 ± 75.0	109.3 ± 93.0	114.0 ± 107.1	121.8 ± 123.3	0.000
Coronary artery disease (*n*, %)	36.4	32.8	42.6	57.3	57.8	0.000
1 coronary artery disease (*n*, %)	1 (4.5)	21 (5.7)	12 (6.8)	12 (7.0)	22 (10.1)
2 coronary artery disease (*n*, %)	2 (9.1)	28 (7.7)	15 (8.5)	10 (5.8)	25 (11.5)
3 coronary artery disease (*n*, %)	5 (22.7)	71 (19.4)	48 (27.3)	76 (44.4)	79 (36.2)
Left main stem stenosis >50 (*n*, %)	1 (4.5)	17 (4.6)	12 (6.8)	11 (6.4)	22 (10.1)	0.430
Ejection Fraction (*n*, %)	57.2 ± 11.6	56.5 ± 11.9	57.5 ± 10.5	56.9 ± 11.0	55.3 ± 12.8	0.224
CCS III or IV (*n*, %)	5 (22.7)	39 (10.7)	25 (14.2)	33 (19.3)	39 (17.9)	0.003
NYHA III or IV (*n*, %)	11 (50.0)	130 (35.5)	66 (37.5)	56 (32.7)	80 (36.7)	0.679
Urgency						0.270
Emergency (*n*, %)	9 (40.9)	51 (13.9)	28 (15.9)	17 (9.9)	28 (12.8)
Urgent (*n*, %)	1 (4.5)	28 (7.7)	14 (8.0)	23 (13.5)	26 (11.9)
Logistic EuroSCORE	22.4 ± 24.9	13.7 ± 16.9	15.9 ± 17.7	12.2 ± 13.9	16.8 ± 19.6	0.623

*Note that the p-value refers to the testing for a non-parametric trend among peak cTnT occurrence groups. PCI, percutaneous coronary intervention; CCS, Canadian Cardiovascular Society classification for angina pectoris; NYHA, New York Heart Association classification for cardiac insufficiency*.

**Table 2B:** Procedure characteristics according to the post-operative period of cTnT peak occurrence in patients with elevated cTnT course (≥1 ng/mL).

	**Period of cTnT peak occurrence**				
	**≤6 h**	**>6 to ≤12 h**	**>12 to ≤18 h**	**>18 to ≤24 h**	**>24 h**	***p***
	**(*n* = 22)**	**(*n* = 366)**	**(*n* = 176)**	**(*n* = 171)**	**(*n* = 218)**
Isolated CABG (*n*, %)	2 (9.1)	35 (9.6)	25 (14.2)	49 (28.7)	53 (24.3)	0.000
Number of distal anastomoses	1.8 ± 1.2	2.6 ± 2.0	2.6 ± 1.7	2.8 ± 1.4	2.5 ± 1.4	0.127
Isolated aortic valve (*n*, %)	3 (13.6)	40 (10.9)	16 (9.1)	14 (8.2)	16 (7.3)	0.028
Isolated mitral valve (*n*, %)	3 (13.6)	51 (13.9)	15 (8.5)	15 (8.8)	12 (5.5)	0.008
CABG & valve (*n*, %)	6 (27.3)	65 (17.8)	36 (20.5)	38 (22.2)	47 (21.6)	0.270
Other operation (*n*, %)	8 (36.4)	175 (47.8)	84 (47.7)	55 (32.2)	90 (41.3)	0.007
Duration of operation (min)	286.0 ± 97.5	265.0 ± 85.0	278.6 ± 93.5	233.3 ± 80.1	252.7 ± 92.1	0.000
ECC time (min)	152.0 ± 49.2	147.6 ± 58.5	148.1 ± 64.8	116.0 ± 52.5	126.1 ± 63.6	0.000
Cross clamp time (min)	98.5 ± 35.3	97.4 ± 40.7	97.4 ± 41.8	76.2 ± 35.2	80.9 ± 38.5	0.000
Defibrillation (*n*, %)	7 (31.8)	146 (39.9)	69 (39.2)	60 (35.1)	64 (29.4)	0.055

*Note that the p-value refers to the testing for a non-parametric trend among peak cTnT occurrence groups. CABG, coronary artery bypass grafting; ECC, extra corporeal circulation*.

**Table 2C:** Post-operative outcomes according to the post-operative period of cTnT peak occurrence in patients with elevated cTnT course (≥1 ng/mL).

	**Period of cTnT peak occurrence**				
	**≤6 h**	**>6 to ≤12 h**	**>12 to ≤18 h**	**>18 to ≤24 h**	**>24 h**	***p***
	**(*n* = 22)**	**(*n* = 366)**	**(*n* = 176)**	**(*n* = 171)**	**(*n* = 218)**
Max. cTnT value (ng/ml)^*^	2.2 (1.6–2.9)	1.7 (1.6–1.8)	2.1 (1.9–2.3)	1.9 (1.7–2.1)	2.1 (1.9–2.4)	0.000
Max. CK-MB value (μg/L)^*^	66 (46–94)	44 (41–48)	60 (53–69)	63 (56–72)	72 (64–81)	0.000
Myocardial infarction (*n*, %)	3 (13.6)	25 (6.8)	22 (12.5)	38 (22.2)	52 (23.9)	0.000
Resuscitation (*n*, %)	0 (0.0)	12 (3.3)	10 (5.7)	6 (3.5)	10 (4.6)	0.437
Stroke (*n*, %)	2 (9.1)	23 (6.3)	15 (8.5)	8 (4.7)	27 (12.4)	0.021
Death (*n*, %)	1 (4.5)	16 (4.4)	11 (6.3)	6 (3.5)	25 (11.5)	0.660
MACCE (*n*, %)	6 (27.3)	54 (14.8)	43 (24.4)	45 (26.3)	93 (42.7)	0.000
ICU stay (d)^*^	1.9 (1.3–2.7)	1.5 (1.4–1.6)	1.7 (1.5–2.0)	1.5 (1.3–1.7)	2.2 (1.9–2.5)	0.000
ICU stay >48 h (*n*, %)	9 (40.9)	107 (29.2)	65 (36.9)	47 (27.5)	110 (50.5)	0.000
New AF (*n*, %)	4 (18.2)	89 (24.3)	45 (25.6)	45 (26.3)	65 (29.8)	0.047
Max. creatinine value (μmol/L)^*^	83 (71–99)	91 (87–95)	101 (94–109)	107 (98–116)	116 (107–126)	0.000
New renal insufficiency or new dialysis (*n*, %)	2 (9.1)	24 (6.6)	21 (11.9)	19 (11.1)	52 (23.9)	0.000
Length of stay (d)^*^	11 (8–15)	11 (10–12)	11 (10–12)	11 (10–12)	13 (11–14)	0.000

Note that the p-value refers to the testing for a non-parametric trend among peak cTnT occurrence groups.

* *Calculations are based on the geometric mean and reference ranges. MACCE, major adverse cardiovascular or cerebrovascular event (as a composite of all-cause mortality, myocardial infarction, or stroke); ICU, intensive care unit*.

All parameters that appeared in this univariate analysis as differently represented between the early vs. late peak of cTnT, as well as logitic EuroSCORE were considered for a multivariable analysis, that was thus conducted to identify the pre-operative and operative parameters, which could have had an influence on the time of peak occurrence (Table 3). It appeared that age (OR: 1.019) and isolated CABG (OR: 1.779) independently influenced the occurrence of a late elevation of cTnT over 1 ng/ml, whereas isolated valve procedures (OR: 0.685) and cross-clamp time (OR: 0.993) independently influenced the occurrence of an early elevation over 1 ng/ml. Interestingly, parameters which independently influenced the late increase of cTnT were slightly different in patients whose cTnT remained below 1 ng/ml (Table 3). Among them, age (OR: 1.023), previous cardiac surgery (OR: 1.562), pre-operative renal insufficiency (OR: 1.402), and logistic EuroSCORE (OR: 1.014) had a highly significant effect (*p* < 0.005).

**Table 3:** Association of preoperative and operative risks with late (>12 h) occurrence of a peak TnT from multivariable analysis, stratified by high (≥1 ng/mL) vs. normal cTnT.

	**cTnT < 1 ng/mL (*****n*** **= 4,193)**		**cTnT ≥1 ng/mL (*****n*** **= 953)**	
	**OR (CI)**	***p***	**OR (CI)**	***p***
Age	1.023 (1.016–1.030)	0.000	1.019 (1.007–1.031)	0.002
Diabetes	1.054 (0.888–1.252)	0.547	1.175 (0.805–1.713)	0.403
Previous cardiac surgery	1.562 (1.159–2.104)	0.003	1.267 (0.829–1.936)	0.274
preop. renal insufficiency	1.402 (1.154–1.704)	0.001	1.042 (0.741–1.465)	0.812
coronary artery disease	1.159 (0.956–1.404)	0.133	1.127 (0.810–1.569)	0.477
Logistic EuroSCORE	1.014 (1.007–1.021)	0.000	1.002 (0.993–1.011)	0.672
Isolated CABG	0.810 (0.663–0.991)	0.041	1.779 (1.114–2.839)	0.016
Isolated valve procedure	1.024 (0.830–1.264)	0.824	0.685 (0.471–0.998)	0.049
Cross clamp time	1.003 (1.000–1.006)	0.030	0.993 (0.990–0.997)	0.001

*CABG, coronary artery bypass grafting*.

Outcomes also varied between patients with early vs. late elevation of cTnT over 1 ng/ml (Tables 2C, 4A and 4B). Complications, and especially PMI but also resuscitation, stroke, death, MACCE or new development of a renal insufficiency were all significantly associated with both early and late peak of post-operative cTnT. However, late cTnT peak (>12 h) was associated with a higher rate of PMI (19.8 vs. 7.2%), death (7.4 vs. 4.4%), MACCE (32.0 vs. 15.5%) or incidence of a new renal insufficiency (16.3 vs. 6.7%).

**Table 4A:** Univariable association of high peak TnT (≥1 ng/mL) with post-operative complications, stratified by early TnT peak.

	**Peak reached ≤12 h after surgery**		
	**<1 ng/mL**	**≥1 ng/mL**	**OR (CI)**
	***n* (2,978)**	***n* (388)**
Myocardial infarction (*n*, %)	4 (0.1)	28 (7.2)	58.2 (20.3–166.8)
Resuscitation (*n*, %)	7 (0.2)	12 (3.1)	13.7 (5.3–34.9)
Stroke (*n*, %)	79 (2.7)	25 (6.4)	2.5 (1.6–4.0)
Death (*n*, %)	5 (0.2)	17 (4.4)	27.2 (10.0–74.3)
MACCE (*n*, %)	86 (2.9)	60 (15.5)	6.2 (4.3–8.7)
ICU stay >48 h (*n*, %)	342 (11.5)	116 (29.9)	3.3 (2.6–4.2)
New renal insufficiency (*n*, %)	50 (1.7)	26 (6.7)	4.2 (2.6–6.8)

*The p-value of each association is ≤ 0.001. MACCE, major adverse cardiovascular or cerebrovascular event (as a composite of all-cause mortality, myocardial infarction, or stroke); ICU, intensive care unit*.

**Table 4B:** Univariable association of high peak TnT (≥1 ng/mL) with post-operative complications, stratified by late TnT peak.

	**Peak reached >12 h after surgery**		
	**<1 ng/mL**	**≥1 ng/mL**	**OR (CI)**
	***n* (1,215)**	***n* (565)**
Myocardial infarction (*n*, %)	9 (0.7)	112 (19.8)	33.3 (16.8–66.3)
Resuscitation (*n*, %)	13 (1.1)	26 (4.6)	4.5 (2.3–8.8)
Stroke (*n*, %)	56 (4.6)	50 (8.8)	2.0 (1.4–3.0)
Death (*n*, %)	19 (1.6)	42 (7.4)	5.1 (2.9–8.8)
MACCE (*n*, %)	79 (6.5)	181 (32.0)	6.8 (5.1–9.0)
ICU stay >48 h (*n*, %)	239 (19.7)	222 (39.3)	2.6 (2.1–3.3)
New renal insufficiency (*n*, %)	71 (5.8)	92 (16.3)	3.1 (2.3–4.3)

*The p-value of each association is ≤ 0.001. MACCE, major adverse cardiovascular or cerebrovascular event (as a composite of all-cause mortality, myocardial infarction, or stroke); ICU, intensive care unit*.

## Discussion

In 2000, Carrier et al. suggested that peak of cTnT following a CABG procedure, occurs around 4–7 h post-reperfusion in a subgroup of patients who will not develop a PMI. Conversely, patients who eventually developed a complication, including a PMI, presented post-operative values of cTnT that kept increasing with a peak occurring between 24 and 48 h post-chest closure (14). This observation was later confirmed by others (19–21). In the current analysis, we approached the patients not from the side of their complication but from the side of their post-operative cTnT profile. We found that several groups can be defined according to the time of the peak occurrence. Approximately 40% of the patients with a post-operative elevation of cTnT over 1 ng/ml had their cTnT peak occurring before 12 h whereas 60% had a delayed increase of cTnT with a peak that occurred later than 12 h. Various time of peak occurrence were also found for those patients with a maximal post-operative cTnT value that remained below 1 ng/ml. Among these later patients, we found that patient's age, history of previous cardiac surgery, pre-operative renal insufficiency and EuroSCORE were independently associated with a late increase of TnT. However, only age independently predicted a late increase over 1 ng/ml whereas the duration of cross-clamp time had an influence on the early increase of cTnT values. Interestingly, isolated CABG procedures were independently associated to a late increase of cTnT whereas isolated valve procedures were associated to an early peak of cTnT.

In fact, several factors have been demonstrated or suggested to influence the post-operative elevation of cTnT values. These include factors related to the surgical procedure as well as factors related to the patient himself. For instance, the duration of cross-clamp time, especially when prolonged over 60 min, is well-known to increase the post-operative values of cTnT (3–7, 12, 23). In addition, the results of the current study seem to indicate that this elevation occurs rather in the early post-operative hours. The type of surgery also seems to play a role. Indeed, cTnT after isolated CABG procedures are not as elevated as after valve surgery or interventions combining CABG and valve surgery. Nesher et al. (1) reported a cTnT value of 0.9 ± 1.5, 1.2 ± 2.9, and 1.3 ± 1.2 ng/ml after isolated CABG, valve procedures or combination of both, respectively (*n* = 1,515, 229, and 174, respectively). Lurati Buse (2) recently reported TnT values of 0.38 ng/ml (IQR: 0.20–0.71) vs. 0.55 ng/ml (0.31–1.07) after isolated CABG vs. other procedures. The reason why we observed an early peak of cTnT after valve surgery as opposed to a later peak after isolated CABG procedures is not clear but may reflect a better cardioplegic protection in CABG vs. valve patients and would fit to the previously mentioned higher level of post-operative cTnT after valve surgery. In fact, valve surgery candidates typically present a left ventricular hypertrophy, i.e., an increase of their cardiac muscle mass, which in turn could proportionally increase the post-operative cTnT value (24–26).

Non-surgical factors also influence the post-operative elevation of cTnT values. Age for instance has recently been shown to be associated with higher cTnT values (27, 28). The pre-operative renal function has also a clear impact on the post-operative cTnT values (9–11). In a very recent study (8) performed in 11,847 patients with chest pain in the emergency room, the authors observed that reduced estimated glomerular filtration rate (eGFR) was the strongest predictor of an elevated troponin level.

A second aspect of our study concerned the clinical relevance of early vs. late increase of cTnT values in terms of prediction of post-operative complications. In fact, we observed that it was rather the elevation over a cut-off value of 1 ng/ml that was determinant. Nevertheless, it also appeared that an early post-operative elevation of cTnT–with a peak value occurring before 12 h—followed by a reduction, was associated with a lower rate of complications, including MI, MACCE and death, as compared to patients with a late increase of cTnT values. This could be explained by the influence of the previously mentioned factors (age, history of previous cardiac surgery, pre-operative renal insufficiency and EuroSCORE) which all have a clear influence on the post-operative outcome. Conversely, early peak of cTnT may rather reflect surgery related factors, such as the duration of ischemia and the quality of the myocardial protection.

The current study has obviously certain limitations including the fact that it is a retrospective study. All data were however prospectively collected, reviewed and completed by a dedicated team of data managers. Another limitation is the choice of a cut-off value of cTnT that is associated with an increased risk of complications. A real cut-off value has indeed not been clearly defined yet however several previous publications, including one from our group, have found a cut-off value around 1 ng/ml, the cut-off value we eventually set, to be reasonably representative of the reality in most cardiac surgery centers (1, 12, 15, 22, 29). Another aspect that was not taken into account is the level of cTnT increase. Although it looks like the average value of the peak was very similar in all time categories, it would be reasonable to expect a higher risk of post-operative complications in patients with higher cTnT values. Finally, the type of ECC and cardioplegia was different for isolated CABG as for other procedures. The combination of MECC and Cardioplexol™ is indeed especially advantageous in isolated CABG and could thus have had an effect on the overall results presented here (30).

As opposed to several articles which focus on specific subgroups of patients, we based the design of our study on the fact that the elevation of cTnT and other markers of perioperative myocardial damage is currently assessed without distinction between subgroups. In that sense, we thought of including a general population of cardiac surgery patients that would also be representative of the activity in most centers. It is true that doing so could have introduced some bias. On the other hand, with a collection of more than five thousands of patients and, we believe, data of high quality, it was possible to run a multivariable analysis including a relatively large series of parameters including subgroups. Finally, it is with this general approach that we could identify some of these subgroups as being associated with different post-operative TnT profiles. For instance isolated CABG procedures appeared related to rather late increase of TnT whereas isolated valve procedures were more related to early peak of TnT. Nevertheless, this information must be interpreted with caution considering the fact that the cut-off value between normal and elevated cTnT groups clearly distinguished two different profiles of patients.

The fact that we cannot provide reliable baseline (pre-operative) values of cTnT and that we cannot totally exclude that some patients had already an elevated starting value prior to surgery can also be seen as a limitation. However, all patients with documented MI during the 8 days prior to surgery have been excluded. In addition and according to recent published papers on cTnT in unstable angina (31), we estimate that even in patients with a possible pre-operative cTnT elevation, this value would most probably still be under 0.1 ng/ml (100 ng/L). For cardiac surgical patients however, it is well-known that the surgical trauma itself contributes to a much higher elevation of cTnT values. In that sense, we estimate that a possible preoperative elevation of cTnT should be considered as neglectable as compared to the inevitable and much higher elevation that follows the surgical trauma. More importantly, we think it is reasonable to estimate that the preoperative cTnT value did not have an influence on the profile of post-operative cTnT elevation.

Our analysis focused on post-operative cTnT. However, several centers use rather troponin I (TnI) or CK-MB as marker of post-operative myocardial damage. We have collected CK-MB values for the same patients and at the same times and although CK-MB was not studied as extensively as cTnT, data displayed in Tables 1C, 2C seem to follow a similar pattern as cTnT. Whereas, similar results would be obtained for TnI cannot be guaranteed and should thus be studied separately. Basically however, values of TnI and cTnT, although not directly proportional tend to follow similar profiles. In that sense, it could reasonably be expected that the results would be similar for TnI.

In conclusion, it seems that besides the severity of post-operative cTnT elevation, the time of peak expression is also of importance. We showed that early peaks of post-operative cTnT values rather reflect difficulties that occurred during the surgical procedure whereas later increases are more related to patients' intrinsic factors. Interestingly, both early and late elevations of cTnT are associated with an increased risk of complications but this risk appears critically higher in case of a late elevation.

## Author Contributions

HT, BG, and VG conceived the original idea and planned the design of the present study. PD, JK, and VG computed the data. HT, PD, JK, BG, and VG analyzed the data. BG and HT performed the statistical analysis. HT wrote the manuscript with the support of all authors. All authors discussed the results and participated to the elaboration of the manuscript.

### Conflict of Interest Statement

The authors declare that the research was conducted in the absence of any commercial or financial relationships that could be construed as a potential conflict of interest.
